# Configurational analysis of ovarian cancer incidence in 30 provinces of China and its policy implications: a fuzzy-set qualitative comparative analysis approach

**DOI:** 10.3389/fpubh.2024.1405010

**Published:** 2024-11-21

**Authors:** Ying Shen, Zhenyu Huang, Chan Li

**Affiliations:** ^1^Department of Obstetrics and Gynecology, Heilongjiang University of Chinese Medicine, Harbin, China; ^2^School of Humanities and Social Sciences, Harbin Engineering University, Harbin, China

**Keywords:** ovarian cancer incidence, environmental-socioeconomic factors, ESE model, FuzzySet qualitative comparative analysis, configuration effect

## Abstract

**Introduction:**

Ovarian cancer is one of the three most common gynecological cancers, with the highest mortality rate among gynecological malignancies. Previous studies on the environmental and socioeconomic (ESE) factors that affect ovarian cancer incidence (OCI) have generally only considered the net effects of single variables, while the synergistic effects among multiple factors have yet to be explored.

**Methods:**

Based on a sample of 30 provinces in Mainland China, an ESE configuration model was constructed in this study, using a fuzzy-set qualitative comparative analysis approach to empirically explore the configuration effects of multiple ESE factors on OCI.

**Results:**

(1) Education, marriage, income, insurance, urbanization, and environment alone do not constitute the necessary conditions for high or low OCI, indicating a need to comprehensively consider the configuration effects of these six conditions. (2) There are two configurations for high OCI: “configuration of environmental pollution under low socioeconomic development” and “configuration of insurance deficiency under high socioeconomic development.” (3) There are two configurations for low OCI: “configuration of insurance adequacy under low socioeconomic development” and “configuration of insurance adequacy under low urbanization.”

**Conclusion:**

The main contribution of this study is its focus on the configuration mechanism of ESE factors, enhancing understanding of the synergistic effects among the multiple factors that affect OCI. The study also provides valuable policy implications for decision-makers to formulate comprehensive health policies for the prevention and treatment of ovarian cancer.

## 1 Introduction

Ovarian cancer (OC) is one of the three most common gynecological cancers, with an incidence that is second only to cervical cancer and breast cancer (1). However, OC has the highest mortality rate among gynecological malignancies, with fewer than 45% of OC patients surviving for 5 years post diagnosis (2). Due to the lack of specific symptoms in the early stages of OC and the lack of effective screening tools in clinical practice, over 70% of OC patients are already in advanced stages of the disease at the time of diagnosis (3). In China, OC has the third highest annual incidence rate of female reproductive system tumors, with the incidence rate having increased by 30% in the past 10 years (4). Thus, OC has become a serious public health issue in China (5, 6).

The main risk factors for OC include genetic and non-genetic factors, with the latter including both clinical and pathological factors, as well as environmental and socioeconomic (ESE) factors (7). However, most studies on the risk factors of ovarian cancer incidence (OCI) only consider genetic or clinical pathological factors: the impact of ESE factors on OCI needs further exploration. Studies have shown that, in addition to genetic factors, different ESE factors have a varied impact on OCI (8). For instance, in terms of environmental factors, research has found heavy metals (9), organic chlorine (10), and endocrine-disrupting chemicals (11) to be associated with various gynecological cancers. The risk of developing OC has been linked with diesel and gasoline engine exhaust gases, as well as with specific industrial toxic emissions (12). In terms of socioeconomic factors, anxiety or depression caused by work and life pressure or lack of social support is related to the incidence and mortality of multiple cancers (13, 14); among the working population, there are significant differences in cancer incidence related to socioeconomic status (15, 16).

However, the existing literature mainly adheres to linear assumptions when studying the impact of ESE factors on OCI, testing the net effect of a single environmental factor or socioeconomic factor on OC incidence, prognosis, or mortality. For instance, Kentros et al. conducted research using general linear regression models, finding that the high level of PM2.5 in the county-level environment was related to 5-year and 10-year OCI (17). In another study, Jiang et al. applied Pearson correlation analysis to investigate the relationship between OC mortality and the Human Development Index (HDI), finding a negative correlation between OC mortality and HDI (18). However, even considering genetic or clinical pathological factors, OCI is an outcome resulting from the complex interactions among ESE factors (19), which requires further exploration. Failure to recognize this complexity makes it challenging to explain why a single variable (such as income or education) is sometimes positively correlated with OCI or OC prognosis, while at other times it is not correlated or has no significant impact (20). Therefore, in studies that consider the net effects of a single variable based on linear assumptions, it is difficult to fully characterize the relationship between ESE factors and OCI; moreover, it also impedes obtaining a clear understanding of the interactions among the different factors.

With theoretical insights from a configuration perspective, the current study adopted a fuzzy-set qualitative comparative analysis (fsQCA) approach to conduct empirical research in response to the aforementioned research gaps in the existing literature. The configuration perspective has been widely applied to understand causal complexity (21). This perspective highlights the interdependency of multiple factors, which can achieve a common goal of influencing outcomes through different permutations and combinations. In this regard, this study incorporated multiple ESE factors into an ESE configuration model. Moreover, the fsQCA approach is suitable for configuration problems and can directly analyze the interdependence among variables (22). Thus, this study used the fsQCA approach to empirically analyze the configuration relationship between ESE factors and OCI. The study aimed to address the following research questions: What are the specific configurations of multiple ESE factors synergistically acting on OCI in China? What are the policy implications of the research findings for China in terms of reducing OCI? The possible contributions of this study are: (1) development of an ESE configuration model capable of comprehensively analyzing the complex causal relationships between multiple ESE factors and OCI; (2) using the fsQCA approach to reveal the configuration mechanisms and types that influence OCI, which may provide systematic policy implications for decision-makers.

The remainder of this paper is organized as follows. Section 2 is an introduction to the materials and methods, as well as the construction of the ESE configuration model. Section 3 presents the results of the fsQCA analysis. Section 4 is the discussion, while Section 5 is the conclusion.

## 2 Materials and methods

### 2.1 Fuzzy-set qualitative comparative analysis approach

We applied fsQCA to analyze the causal relationship between ESE factors and OCI. Qualitative comparative analysis (QCA) is an asymmetric research method that focuses on configuration effects and is based on techniques such as set theory and Boolean algebra. QCA combines the advantages of quantitative and qualitative methods, focusing on the complex causal relationships and causal asymmetry between conditions and outcomes (23). The fsQCA approach, which is suitable for processing continuous data, is one of the method categories of QCA. This approach is suitable for studies with samples of more than 100 subjects and also for studies with samples less than 50 subjects (24).

The fsQCA approach is applicable to the current study because it focuses on the causal relationships between multiple ESE factors and OCI, which is a configuration issue. The existing literature shows that OCI is influenced by multiple ESE factors. However, different regions have their own characteristic factor composition and utilization; thus, focusing only on a particular factor means it is difficult to explain the differences in OCI in different regions. Moreover, the ESE factors are interdependent and mutually influential, potentially acting on a specific OCI outcome through differentiated configuration matching. For example, in an area with relatively high income and urbanization, pollution in that area may also be relatively severe, which in turn can jointly lead to an outcome of high OCI. In addition, the 30 provinces analyzed in this study meet the sample requirements of fsQCA (24).

### 2.2 Variables and data source

#### 2.2.1 ESE configuration model

**Figure 1:**
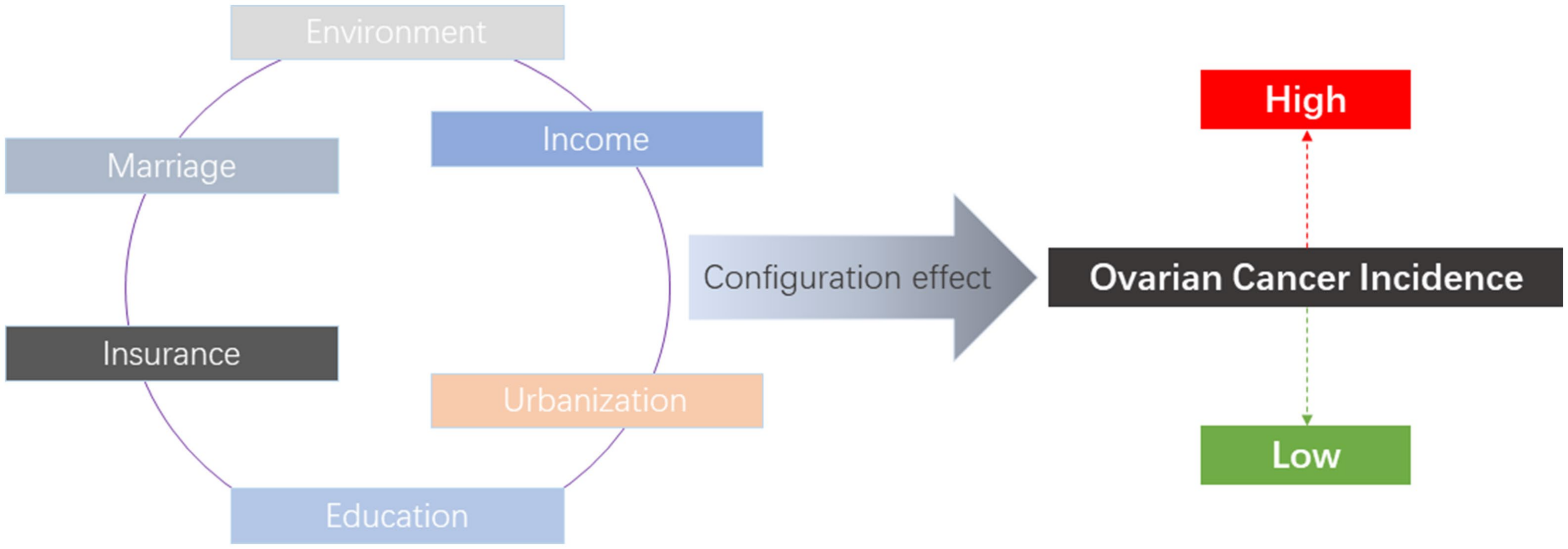
ESE configuration model for OCI.

The fsQCA approach requires a balance between the number of conditions and the number of cases, meaning that for samples of no more than 50 subjects, the number of conditions is generally around 6 (23). Thus, OCI was taken as the outcome variable and an ESE configuration model was constructed (see Figure 1).

With OCI as the outcome variable, the ESE configuration model is composed of education, marriage, income, insurance, urbanization, and environment as the condition variables. Among them, education, marriage, income, insurance, and urbanization are socioeconomic factors, while the environmental factor mainly refers to the pollution from industrial emissions in specific regions affecting the environment. Education (25), marriage (26), income (27), insurance (28), and urbanization (29) are factors that have been extensively explored in the existing OCI research literature, but they are also factors that have not yielded clear conclusions. Some studies have shown a significant correlation between the aforementioned factors and OCI, while others have not found a significant correlation. However, a correlation between environmental pollution and OCI has been confirmed (9–12, 18). As important factors associated with human beings, environmental factors coexist with socioeconomic factors. Therefore, environmental and socioeconomic factors have been integrated into the ESE configuration model to deepen understanding of the relationship between OCI and non-genetic and non-clinical factors.

#### 2.2.2 Condition and outcome variables

The data in this study came from 30 provinces in the Chinese Mainland in 2019 (Tibet was not included in the sample due to missing data. This study also does not include data from Hong Kong, Macao, and Taiwan in China). The reasons for selecting the year 2019 are as follows: 2020–2022 were the 3 years of the global COVID-19 pandemic, and also the years when the Chinese government implemented strict prevention and control measures that suspended most socioeconomic activity. Therefore, the data during this period has a certain specificity and lacks universal reference value. As of the completion of this manuscript, the statistical report for 2023 has not yet been released. The variable settings, measurement, and data sources of this study are shown in Table 1.

(1) Outcome. The OCI of each province was used as an outcome indicator of outcome, and the data was sourced from the *China Statistical Yearbook of Health*.
(2) For socioeconomic factors, we measured Education (x1) using the proportion of women with high school education and above to the population aged-6 and above in each province, and the data was sourced from the *Educational Statistics Yearbook of China*. The divorce rate of each province was used to measure Marriage (x2), the *per capita* disposable income by province to measure Income (x3), and the proportion of urban population to total population in each province to measure Urbanization (x5), and the data was sourced from the *China Statistical Yearbook*. Insurance (x4) was measured using the proportion of basic medical insurance participants to the total number of people in each province, and the data was sourced from the *China Statistical Yearbook of Health*.
(3) For Environment (x6), completed investment in treatment of industrial pollution/total population by province was used for measurement, and the data was sourced from the *China Statistical* Yearbook *of Environment*. An important reason for adopting this measurement method is that in the current political environment in China where green economic development is highly valued (30), local governments have regarded environmental governance as a political task. Therefore, from the data in the *China Statistical Yearbook of Environment*, provinces with more severe environmental pollution have higher completed investment in the treatment of industrial pollution; thus, this can effectively reflect the overall industrial pollution situation of the province in the corresponding year.

**Table 1:** Variables, indicators, measurement, and data sources.

Variables	Indicators	Measurement	Sources
Outcome	OCI (y)	Ovarian cancer incidence by province	*China Statistical Yearbook of Health*
Conditions	Education (x1)	Women with high school education and above/population aged 6 and above by province	*Educational Statistics Yearbook of China*
	Marriage (x2)	Divorce rate by province	*China Statistical Yearbook*
	Income (x3)	*Per capita* disposable income by province	*China Statistical Yearbook*
	Insurance (x4)	Number of participants with basic medical insurance/total population by province	*China Statistical Yearbook of Health*
	Urbanization (x5)	Urban population/total population by province	*China Statistical Yearbook*
	Environment (x6)	Completed investment in treatment of industrial pollution/total population by province	*China Statistical Yearbook of Environment*

### 2.3 Calibration

The initial conditions and outcome data of this study were sourced from authoritative reports but lacked an external basis and theoretical standards for calibration. In order to determine the set membership of the provinces in the samples, the direct calibration method was used to calibrate the initial data (22, 23). The calibration points of Fully in, Crossover, and Fully out of the variables were set to 75, 50, and 25% of the descriptive statistical values of the samples, respectively. The initial data was then transformed into set membership values ranging from 0 to 1. The calibration points for each variable are shown in Table 2.

**Table 2:** Calibration points.

Outcome and conditions	Calibration points		
	Fully in	Crossover	Fully out
y	3.125	1.750	1.000
x1	0.332	0.299	0.266
x2	0.039	0.034	0.028
x3	32,768.800	26,338.750	23,884.150
x4	1.023	0.949	0.911
x5	0.671	0.595	0.551
x6	74.757	37.309	17.496

The software fsQCA 4.0 was adopted to process data. Due to the existence of a situation where the value of the crossover was exactly 0.5 after calibration, we adjusted 0.5–0.499 or 0.501 by ±0.001 (31). Table 3 shows the results of the calibration.

**Table 3:** Results of calibration.

Province	y	x1	x2	x3	x4	x5	x6
Beijing	0.03	1	0.95	1	0.67	1	0.01
Tianjin	1	1	1	1	0	1	0.97
Hebei	0.53	0.07	0.501	0.3	0.06	0.22	0.72
Shanxi	0.05	0.93	0.01	0.04	0	0.499	1
Inner Mongolia	0.05	1	0.95	0.88	0	0.82	0.99
Liaoning	1	0.95	0.99	0.93	0.01	0.97	0.19
Jilin	0.05	0.41	1	0.1	0.46	0.3	0.14
Heilongjiang	0.86	0.59	1	0.07	0	0.63	0.01
Shanghai	0.84	1	0.01	1	0	1	1
Jiangsu	0.58	0.96	0.86	1	0.73	0.99	0.95
Zhejiang	0.02	0.78	0.02	1	0.23	0.98	0.84
Anhui	0.45	0	0.95	0.51	0.99	0.07	0.6
Fujian	1	0.01	0.05	0.99	0.54	0.94	0.31
Jiangxi	0.86	0.09	0.05	0.48	0.96	0.19	0.61
Shandong	0.35	0.07	0.05	0.92	0.51	0.69	0.99
Henan	0.95	0.1	0.86	0.05	0.99	0.01	0.63
Hubei	1	0.97	0.77	0.72	0.31	0.64	0.1
Hunan	0.07	0.96	0.27	0.65	0.71	0.17	0.01
Guangdong	0.35	0.93	0	1	0.26	0.99	0.19
Guangxi	0.02	0.01	0.08	0.02	0.98	0	0.02
Hainan	0.07	0.78	0	0.54	0.73	0.45	0.01
Chongqing	1	0.84	1	0.77	0.98	0.95	0.02
Sichuan	0.53	0.12	0.92	0.12	0.96	0.02	0.03
Guizhou	0.07	0	1	0	1	0	0.13
Yunnan	0.63	0	0.12	0.01	0.22	0	0.13
Shaanxi	0.03	0.71	0.65	0.11	0.95	0.49	0.96
Gansu	0.02	0.02	0.01	0	0.72	0	0.05
Qinghai	1	0.01	0.12	0.01	0.08	0.06	0.89
Ningxia	0.2	0.23	0.65	0.09	0.05	0.54	0.99
Xinjiang	0.96	0.41	0.08	0.02	0.04	0.01	0.85

## 3 Results

### 3.1 Necessity analysis

According to the fsQCA analysis steps, before conducting sufficiency analysis it should be tested whether a single condition variable constitutes a necessary condition for high OCI (y) or low OCI (~y). The value of consistency is an important indicator for testing whether a single condition variable constitutes a necessary condition. When the consistency of a condition variable is less than 0.9, it can be considered that this condition variable did not constitute a necessary condition for the outcome (24). As shown in Table 4, the consistency of all condition variables is lower than 0.9, indicating that none of the condition variables constitute a necessary condition for explaining the outcome. These results indicate that the synergistic effects of the six condition variables on OCI require comprehensive consideration.

**Table 4:** Results of necessity analysis.

Conditions	y	~y
	Consistency	Consistency
x1	0.522	0.553
~ x1	0.559	0.524
x2	0.589	0.508
~x2	0.514	0.589
x3	0.584	0.485
~x3	0.530	0.623
x4	0.456	0.593
~x4	0.658	0.515
x5	0.577	0.500
~x5	0.525	0.596
x6	0.564	0.500
~x6	0.545	0.603

As with the QCA approach, the symbol “~” means Not (or not high, low, weak, etc.) in set operation, e.g., x1 means well educated (or “high education” in this study), while ~ y means undereducated (or “low education” in this study).

### 3.2 Sufficiency analysis

#### 3.2.1 Standard analyses

**Figure 2:**
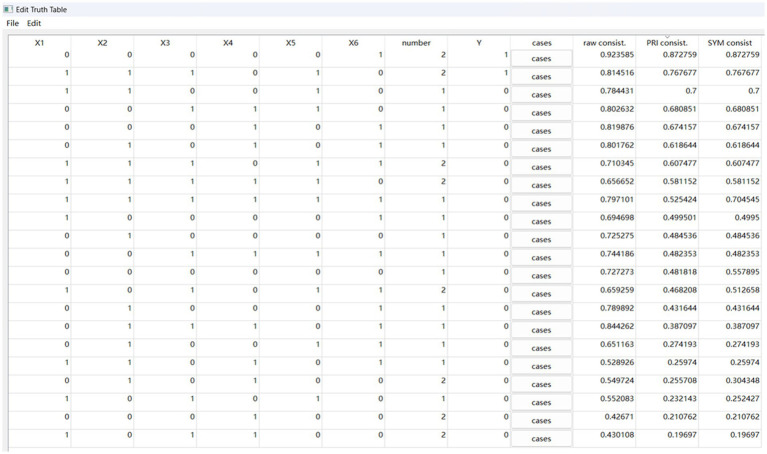
Truth table for y.

**Figure 3:**
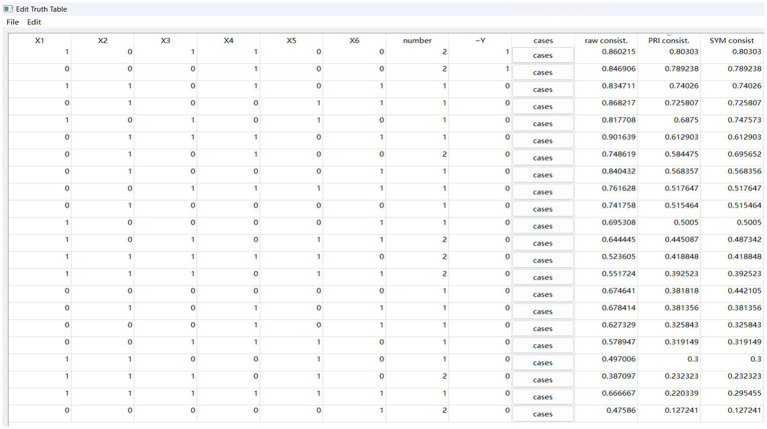
Truth table for ~y.

**Figure 4:**
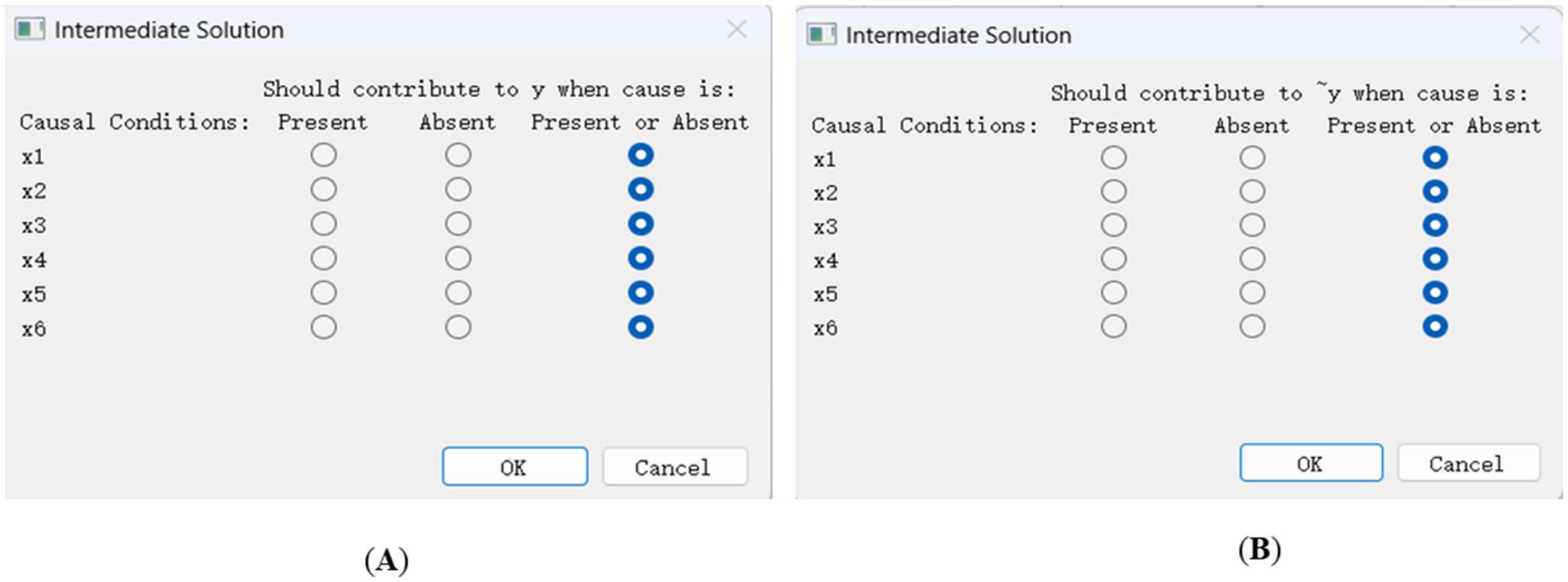
Intermediate solution program. **(A)** Choosing how the causal conditions contribute to y. **(B)** Choosing how the causal conditions contribute to ~y.

The purpose of sufficiency analysis is to obtain different configurations composed of multiple conditions that lead to the outcomes. In the fsQCA4.0 software, sufficiency analysis is carried out through standard analyses programs. According to references (21–23), the operations for standard analyses are as follows:

(1) Parameter settings. To distinguish whether the configuration has passed the consistency test, the consistency threshold is set to 0.8. Considering that the total number of cases in this study is 30, the frequency threshold should be set to 1. To reduce potential conflicting configurations, the value of proportional reduction in inconsistency (PRI) was set to 0.75. Then we obtained the truth table for y (see Figure 2) and the truth table for ~y (see Figure 3).
(2) Option settings for standardized analysis of y and ~ y. When conducting the standard analyses of y and ~ y, as shown in Figure 4, according to the results of the necessity analysis none of the condition variables can constitute a necessary condition. Thus, the “present or absent” option was selected in the intermediate solution program.
(3) Complex solutions, parsimonious solutions, and intermediate solutions for y and ~ y (see Table 5). The condition that appears simultaneously in the intermediate solution and the parsimonious solution is the core condition, and the condition that only appears in the intermediate solution and does not appear in the parsimonious solution is the peripheral condition.

**Table 5:** Configuration solution for y and ~ y.

Solutions	Configurations
Configuration Solution for y	
Complex Solution	~x1* ~ x2* ~ x3* ~ x4* ~ x5*x6
	x1*x2*x3* ~ x4*x5* ~ x6
Parsimonious Solution	~x1* ~ x2* ~ x4*x6
	x2*x3* ~ x4* ~ x6
Intermediate Solution	~x1* ~ x2* ~ x3* ~ x4* ~ x5*x6
	x1*x2*x3* ~ x4*x5* ~ x6
Configuration Solution for ~y	
Complex solution	~x1* ~ x2* ~ x3*x4* ~ x5*x6
	x1* ~ x2*x3*x4* ~ x5* ~ x6
Parsimonious Solution	~x2*x4* ~ x5* ~ x6
Intermediate Solution	x1* ~ x2* ~ x3* ~ x4* ~ x5*x6
	~x1*x2* ~ x3*x4*x5*x6

The symbol * is generated by the software when running the standard analyses, meaning the interaction among different conditions.

The interpretation of the sufficiency analysis results mainly depends on the parsimonious solution and the intermediate solution (32, 33). We present the results in the format developed by Ragin and Fiss (see Table 6) (34).

**Table 6:** Results of sufficiency analysis.

Conditions	y		~y	
	H1	H2	L1	L2
x1	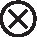	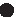	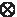	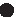
x2	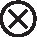	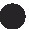	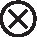	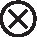
x3	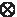	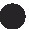	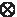	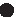
x4	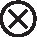	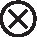	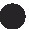	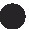
x5	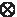	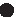	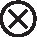	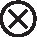
x6	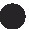	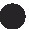	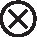	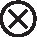
Raw coverage	0.182	0.139	0.169	0.104
Unique coverage	0.164	0.120	0.128	0.063
Solution coverage	0.303		0.231	
Solution consistency	0.868		0.842	

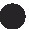
 (big solid circle) means the presence of the core condition, 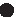 (small solid circle) means the presence of the peripheral condition, 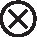 (big circle with × in it) means the absence of the core condition, 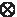 (small circle with × in it) means the absence of the peripheral condition, and blank means that the presence or absence of the condition is insignificant to the outcome.

#### 3.2.2 Configurations of y

Table 6 presents two configurations of high OCI (y). The solution consistency is 0.868, meaning that among all samples meeting these two configurations, 86.8% of provinces have high OCI. The solution coverage is 0.303, meaning that the two configurations can explain 30.3% of the provinces with high OCI.

(1) H1: Configuration of Environmental Pollution under Low Socioeconomic Development. In H1, low Education (~x1), low Divorce (~x2), low Insurance (~x4), and high Pollution (x6) are the core conditions, while low Income (~x3) and low Urbanization (~x5) are the peripheral conditions. The combination of these conditions can lead to high OCI (y). According to the combination characteristics, H1 can be named as “Configuration of Environmental Pollution under Low Socioeconomic Development.” H1 can explain about 18.2% of the high-OCI samples, of which about 16.4% can only be explained by this configuration. Two typical provinces of H1 are Qinghai and Xinjiang.
(2) H2: Configuration of Insurance Deficiency under High Socioeconomic Development. In H2, high Divorce (x2), high Income (x3), low Insurance (~x4), and low Pollution (~x6) are the core conditions, while high Education (x1) and high Urbanization (x5) are the peripheral conditions. The combination of these conditions can lead to high OCI (y). According to the combination characteristics, H2 can be named as “Configuration of Insurance Deficiency under High Socioeconomic Development.” H2 can explain about 13.9% of the high-OCI samples, of which about 12% can only be explained by this configuration. Two typical provinces of H2 are Liaoning and Hubei.

#### 3.2.3 Configurations of ~y

Table 6 presents two configurations of low OCI (~y). The solution consistency is 0.842, meaning that among all samples meeting these two configurations, 84.2% of provinces have low OCI. The solution coverage is 0.231, meaning that the two configurations can explain 23.1% of the provinces with low OCI.

(1) L1: Configuration of Insurance Adequacy under Low Socioeconomic Development. In L1, low Divorce (~x2), high Insurance (x4), low Urbanization (~x5), and low Pollution (~x6) are the core conditions, while low Education (~x1) and low Income (~x3) are the peripheral conditions. The combination of these conditions can lead to low OCI (~y). According to the combination characteristics, L1 can be named as “Configuration of Insurance Adequacy under Low Socioeconomic Development.” L1 can explain about 16.9% of the low-OCI samples, of which about 12.8% can only be explained by this configuration. Two typical provinces of L1 are Guangxi and Gansu.
(2) L2: Configuration of Environmental Pollution under Low Socioeconomic Development. In L2, low Divorce (~x2), high Insurance (x4), low Urbanization (~x5), and low Pollution (~x6) are the core conditions, while high Education (x1) and high Income (x3) are the peripheral conditions. The combination of these conditions can lead to low OCI (~y). According to the combination characteristics, L2 can be named as “Configuration of Insurance Adequacy under Low Urbanization.” L2 can explain about 10.4% of the low-OCI samples, of which about 6.3% can only be explained by this configuration. Two typical provinces of L2 are Hunan and Hainan.

## 4 Discussion

### 4.1 Core and peripheral conditions of strong MER configurations

Some of the results of this study are consistent with those in the existing literature when considering only the relationship between a particular single conditional variable and OCI.

(1) Low Education is a core condition of H1, and some studies have confirmed that there is a negative correlation between education and the incidence, prognosis, and other conditions of OC (20).
(2) H1 and H2 suggest there is a negative correlation between Insurance and OCI, one possible reason being that individuals who purchase basic medical insurance have reduced their future stress expectations to a certain extent (13).
(3) High Pollution is a core condition of H1, and this result is consistent with existing research, such as (12) and (17).
(4) High Divorce rate is a core condition for H2, which is consistent with current research on the relationship between marriage and OCI (26).
(5) Low Income is a peripheral condition of H1, and a negative correlation between income and OCI has been confirmed in existing studies, such as (27).
(6) Finally, high Urbanization is a peripheral condition of H2, and the negative consequences of urbanization processes, such as industrial pollution and high living pressure, have been analyzed in the literature [e.g., (29)] for their positive correlation with OCI.

L1 and L2 to some extent confirm the above results. For instance, in configurations L1 and L2, low Divorce, High Insurance, low Urbanization, and low Pollution are the core conditions leading to low OCI; in configuration L2, high Education and high Income are the peripheral conditions leading to low OCI.

However, it is worth noting that in H1, low Income is a core condition and low Urbanization is a peripheral condition; in H2, on the contrary, high Income is a core condition and high Urbanization is a peripheral condition. Similarly, in L1, low Education and low Income are peripheral conditions leading to low OCI; in L2, high Education and high Income are the peripheral conditions leading to low OCI. These results may not be understood if reliant on the explanatory logic of the existing literature. In this regard, it is necessary to understand OCI by shifting the perspective from univariate and linear analysis to configuration effects.

### 4.2 ESE factors and the configuration mechanism of OCI

As pointed out in the introduction, existing studies have investigated the correlation between single variables and OCI under a linear assumption, focusing on the net effects of this single variable. This has led to previous studies not being able to convincingly explain why the same variable is sometimes significantly correlated with OCI, sometimes not [such as marriage (35)], and sometimes even in complete opposition to OCI. An important reason is the neglect of the synergistic effects and configuration effects among ESE factors. The fsQCA approach is suitable for exploring configuration problems and can directly analyze the interdependence among variables (21, 22), thereby helping researchers identify the causal complexity between different combinations of conditions and outcomes, as well as the equivalent substitution effects between conditions. Through empirical analysis using the fsQCA approach from a configuration perspective, this study reveals the different effects of differentiated combinations of ESE factors on OCI.

(1) Horizontal comparison and causal asymmetry. By comparing the four configurations horizontally, it is evident that, unlike the study of linear assumptions, the factors leading to high and low OCI are not necessarily the same variable. For example, the combination of ~x1, ~x2, and ~ x3 in H1 with other factors leads to y, while the combination of ~x1, ~x2, and ~ x3 in L1 with other factors actually leads to ~y. Similarly, the combination of x1 and ~ x6 in H2 with other factors leads to y, while the combination of x1 and ~ x6 in L2 with other factors leads to ~y. This situation results in causal asymmetry (36). In the perspective of set theory, the interdependence and synergistic effects among different factors are widely present in reality (24), leading to complexity in causal relationships. In other words, the impact of a specific ESE factor on OCI depends on its configuration relationship with other ESE factors.
(2) Vertical comparison and equivalent substitution effects. By comparing the four configurations vertically, it is evident that different combinations of the six condition variables constitute two high-OCI configurations and two low-OCI configurations, revealing the existence of equivalent substitution effects between different configurations. On the one hand, comparison of the similarities and differences between H1 and H2 shows that given the same conditional variable “~x4,” there is a substitution effect between “~x1 * ~ x2 * ~ x3 * ~ x5 * x6” and “x1 * x2 * x3 * x5 * ~ x6” (the symbol * represents the interaction and configuration effect among variables). Comparison of the similarities and differences between L1 and L2 shows that, given the same combination of condition variables “~x2 * x4 * ~ x5 * ~ x6,” “~x1 * ~ x3” and “x1 * x3” have substitution effects. The existence of equivalent configurations and substitution effects indicates that the combination of conditions leading to high or low OCI is not unique. This has practical significance for decision-makers in different provinces, enabling them to formulate comprehensive OC prevention and treatment policies that are conducive to reducing OCI.

### 4.3 Policy implications and research limitations

In recent years, OCI in China has shown a significant upward trend, posing a serious threat to women’s lives and health. In the absence of typical clinical features and effective screening methods in the early stage of the disease, studying the causes through ESE factors holds great significance for the prevention and treatment of OC. In this regard, the main policy implications are as follows:

(1) To strengthen the governance of environmental pollution and accelerate the green and low-carbon transformation of socioeconomic development. H1, L1, and L2 indicate that environmental pollution is a core condition affecting OCI. Therefore, for regions with high OCI, government decision-makers need to strengthen their efforts in environmental pollution control and increase corresponding fiscal expenditures. It is necessary to accelerate green and low-carbon technological innovation, optimize industrial structure, and thus accelerate the green and low-carbon transformation of socioeconomic development.
(2) To improve people’s quality of life and formulate policies that are conducive to expanding people’s participation in medical insurance. First, H1 indicates that when socioeconomic development lags behind, environmental pollution is more likely to lead to high OCI. Therefore, while strengthening environmental pollution control, government decision-makers need to formulate social and economic policies that are conducive to promoting high-quality development; Second, H2 suggests that high OCI may also occur in regions with better socioeconomic development, possibly due to work and life stress. When stress levels are generally too high, this can lead to depression and anxiety, which is closely related to high OCI. Therefore, while driving socioeconomic development, the key is to improve people’s quality of life, such as reducing divorce rates, increasing income and a sense of gain. Finally, insurance is an important condition that affects OCI across the four configurations. This indicates that formulating policies conducive to expanding people’s participation in medical insurance is a key measure in reducing OCI.
(3) To attach importance to the synergistic effect of ESE factors and develop comprehensive health policies tailored to local conditions that are beneficial for the prevention and treatment of OC. The necessity analysis showed that none of the six ESE factors can constitute a necessary condition for explaining the outcome alone, and the presence of configuration effects reveals the complexity of OCI. In other words, although we have emphasized the importance of the environment and insurance, government decision-makers cannot simply allocate their attention to a specific factor but must develop comprehensive health policies tailored to local conditions for the prevention and treatment of OC. In practice, decision-makers can generate configuration effects by driving the mutual adaptation of local ESE factors to form effective low OCI policy paths suitable for this region. This requires government decision-makers to make a comprehensive and effective assessment of the ESE factors in this region, dynamically adjusting different influencing factors based on the actual situation. For example, in the regions where the situation may be similar to Guangxi or Gansu, the policy path of “configuration of insurance adequacy under low socioeconomic development” can be chosen. In other regions where the situation may be similar to that of Hunan or Hainan, then the policy path of “configuration of environmental pollution under low socioeconomic development” may be a suitable choice.

Finally, this study has some limitations that need to be addressed in future research.

(1) Although the ESE configuration model used in this study covers six ESE factors, there are still some omissions due to the number of samples. Future improvements are possible by expanding the research focus from China to a global scale and increasing the number of condition variables by increasing the number of samples. Variable dimensionality reduction methods such as factor analysis or principal component analysis could also be used to enable individual variables to accommodate more information.
(2) Qualitative factors should be considered to analysis in the ESE configuration models. On the one hand, we can conduct a global comparative study based on countries, which allows us to incorporate qualitative factors such as culture and institutions in our model; On the other hand, we can conduct a social survey, in which the human behavior, social status, social classes and social mobility etc. can be included in the model.
(3) This study only considered ESE factors, and in future studies genetic and clinical pathological factors could be included in the configuration model to enable a more comprehensive and systematic OCI analysis, providing a more comprehensive scientific basis for the prevention and treatment of OC.
(4) Since this is the first time that we applied QCA approach to the systematic study of OCI, we did not carry out cross year case data analysis, which limited our research in accounting for temporal changes. At present, QCA approach still needs to develop in dealing with variables at different levels such as time. In the future, how to incorporate the temporal changes in the model will be an important direction for the future development of QCA approach, which may help us to further understand the temporal changes between ESE factors and OCI over time.

## 5 Conclusion

This study used a sample of 30 provinces in China, integrating six conditional variables in the construction of an ESE configuration model and empirically analyzing the configuration effect of multiple ESE factors on OCI using the fsQCA approach. Our main research findings are as follows:

(1) Education, Marriage, Income, Insurance, Urbanization, and Environment alone cannot constitute the necessary conditions for high or low OCI, indicating a need to comprehensively consider the configuration effects of these six conditions.
(2) There are two configurations for high OCI: configuration of environmental pollution under low socioeconomic development, and configuration of insurance deficiency under high socioeconomic development.
(3) There are two configurations for low OCI: “configuration of insurance adequacy under low socioeconomic development” and “configuration of insurance adequacy under low urbanization.”

The main contributions of this study may be as follows:

(1) By integrating multiple ESE factors into a configuration model with OCI as the outcome, we may deepen understanding of the complex causal relationship between ESE factors and OCI.
(2) By revealing the configuration types of high and low OCI, and focusing on the configuration mechanism of ESE factors acting on OCI, the study may provide some valuable policy implications for decision-makers to formulate comprehensive health policies for the prevention and treatment of OC.

## Data Availability

The original contributions presented in the study are included in the article/Supplementary material, further inquiries can be directed to the corresponding author.
