# Supplementary material for: Configurational analysis of ovarian cancer incidence in 30 provinces of China and its policy implications: a fuzzy-set qualitative comparative analysis approach
Source: Front Public Health. 2024 Nov 21;12:1405010. doi: 10.3389/fpubh.2024.1405010 (PMC11617518; doi:10.3389/fpubh.2024.1405010)
Supplement: Supplementary file 13 [file Table_13.DOCX]

# Necessity Analysis

## Outcome variable: y

Conditions tested:

Consistency Coverage

X1 0.521620 0.508361

~X1 0.559369 0.541528

X2 0.588950 0.575095

~X2 0.514001 0.496651

X3 0.584077 0.593859

~X3 0.529856 0.492661

X4 0.456417 0.470297

~X4 0.657515 0.604035

X5 0.576527 0.574202

~X5 0.525052 0.497690

X6 0.564173 0.573222

~X6 0.544955 0.507024

## Outcome variable: ~y

Conditions tested:

Consistency Coverage

X1 0.552819 0.570569

~X1 0.523655 0.536877

X2 0.508101 0.525434

~X2 0.589112 0.602825

X3 0.484770 0.521982

~X3 0.622813 0.613274

X4 0.593001 0.647101

~X4 0.514582 0.500631

X5 0.499611 0.526967

~X5 0.596306 0.598595

X6 0.499676 0.537657

~X6 0.603370 0.594508

# Sufficiency Analysis

## y


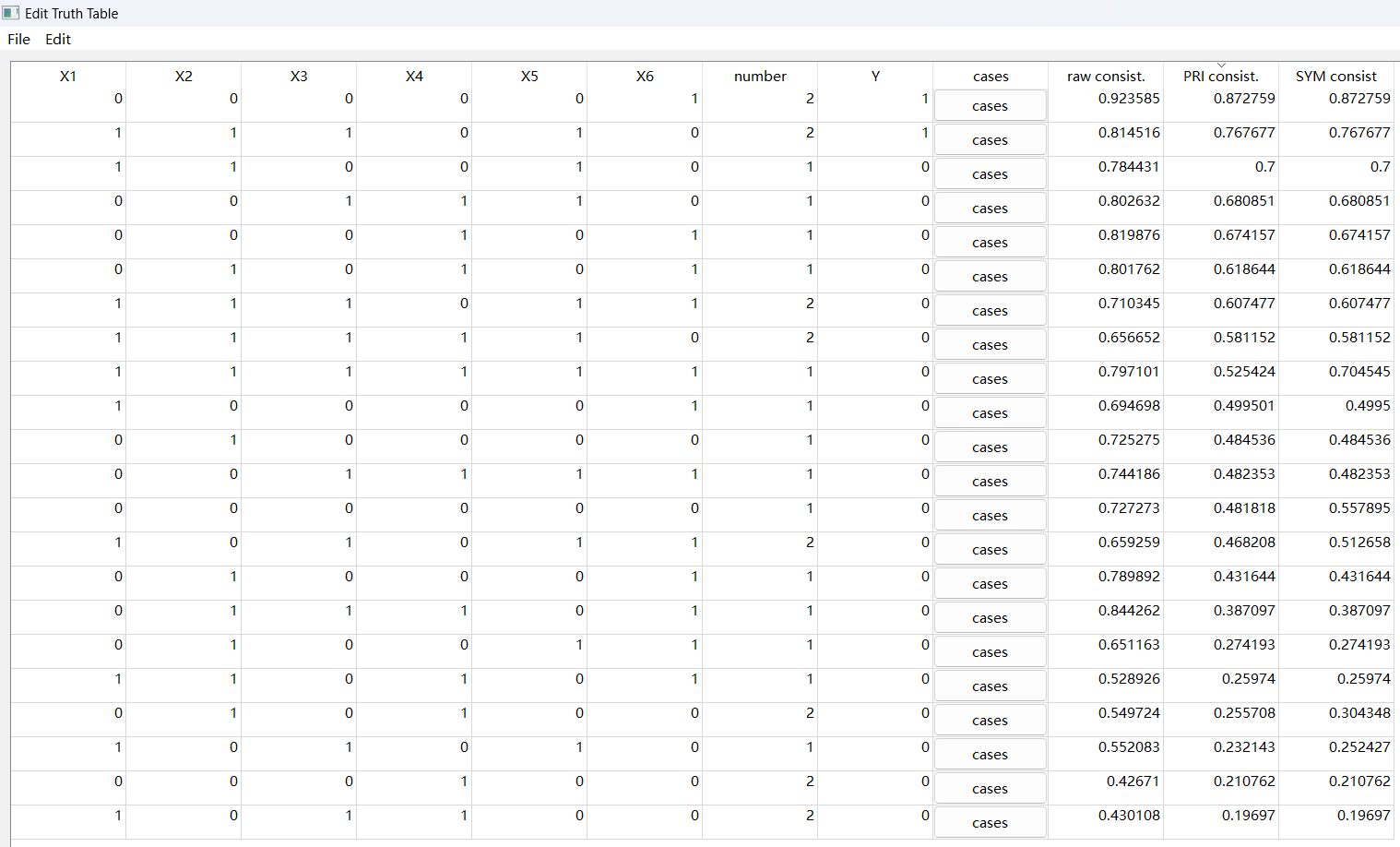


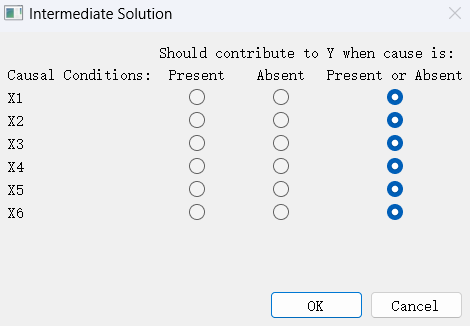


**********************

*TRUTH TABLE ANALYSIS*

**********************

File: D:/桌面/卵巢癌-校准数据-75.csv

Model: Y = f(X1, X2, X3, X4, X5, X6)

Algorithm: Quine-McCluskey

### --- COMPLEX SOLUTION ---

frequency cutoff: 1

consistency cutoff: 0.814516

raw unique

coverage coverage consistency

---------- ---------- ----------

~X1*~X2*~X3*~X4*~X5*X6 0.182498 0.163967 0.923585

X1*X2*X3*~X4*X5*~X6 0.138641 0.12011 0.814516

solution coverage: 0.302608

solution consistency: 0.868084

Cases with greater than 0.5 membership in term ~X1*~X2*~X3*~X4*~X5*X6: Qinghai (0.88,1), Xinjiang (0.59,0.96)

Cases with greater than 0.5 membership in term X1*X2*X3*~X4*X5*~X6: Liaoning (0.81,1), Hubei (0.64,1)

**********************

*TRUTH TABLE ANALYSIS*

**********************

File: D:/桌面/卵巢癌-校准数据-75.csv

Model: Y = f(X1, X2, X3, X4, X5, X6)

Algorithm: Quine-McCluskey

### --- PARSIMONIOUS SOLUTION ---

frequency cutoff: 1

consistency cutoff: 0.814516

raw unique

coverage coverage consistency

---------- ---------- ----------

~X1*~X2*~X4*X6 0.230542 0.186616 0.857106

X2*X3*~X4*~X6 0.164036 0.12011 0.810169

solution coverage: 0.350652

solution consistency: 0.821514

Cases with greater than 0.5 membership in term ~X1*~X2*~X4*X6: Qinghai (0.88,1), Xinjiang (0.59,0.96)

Cases with greater than 0.5 membership in term X2*X3*~X4*~X6: Liaoning (0.81,1), Hubei (0.69,1)

**********************

*TRUTH TABLE ANALYSIS*

**********************

File: D:/桌面/卵巢癌-校准数据-75.csv

Model: Y = f(X1, X2, X3, X4, X5, X6)

Algorithm: Quine-McCluskey

### --- INTERMEDIATE SOLUTION ---

frequency cutoff: 1

consistency cutoff: 0.814516

Assumptions:

raw unique

coverage coverage consistency

---------- ---------- ----------

~X1*~X2*~X3*~X4*~X5*X6 0.182498 0.163967 0.923585

X1*X2*X3*~X4*X5*~X6 0.138641 0.12011 0.814516

solution coverage: 0.302608

solution consistency: 0.868084

Cases with greater than 0.5 membership in term ~X1*~X2*~X3*~X4*~X5*X6: Qinghai (0.88,1), Xinjiang (0.59,0.96)

Cases with greater than 0.5 membership in term X1*X2*X3*~X4*X5*~X6: Liaoning (0.81,1), Hubei (0.64,1)

## ~y


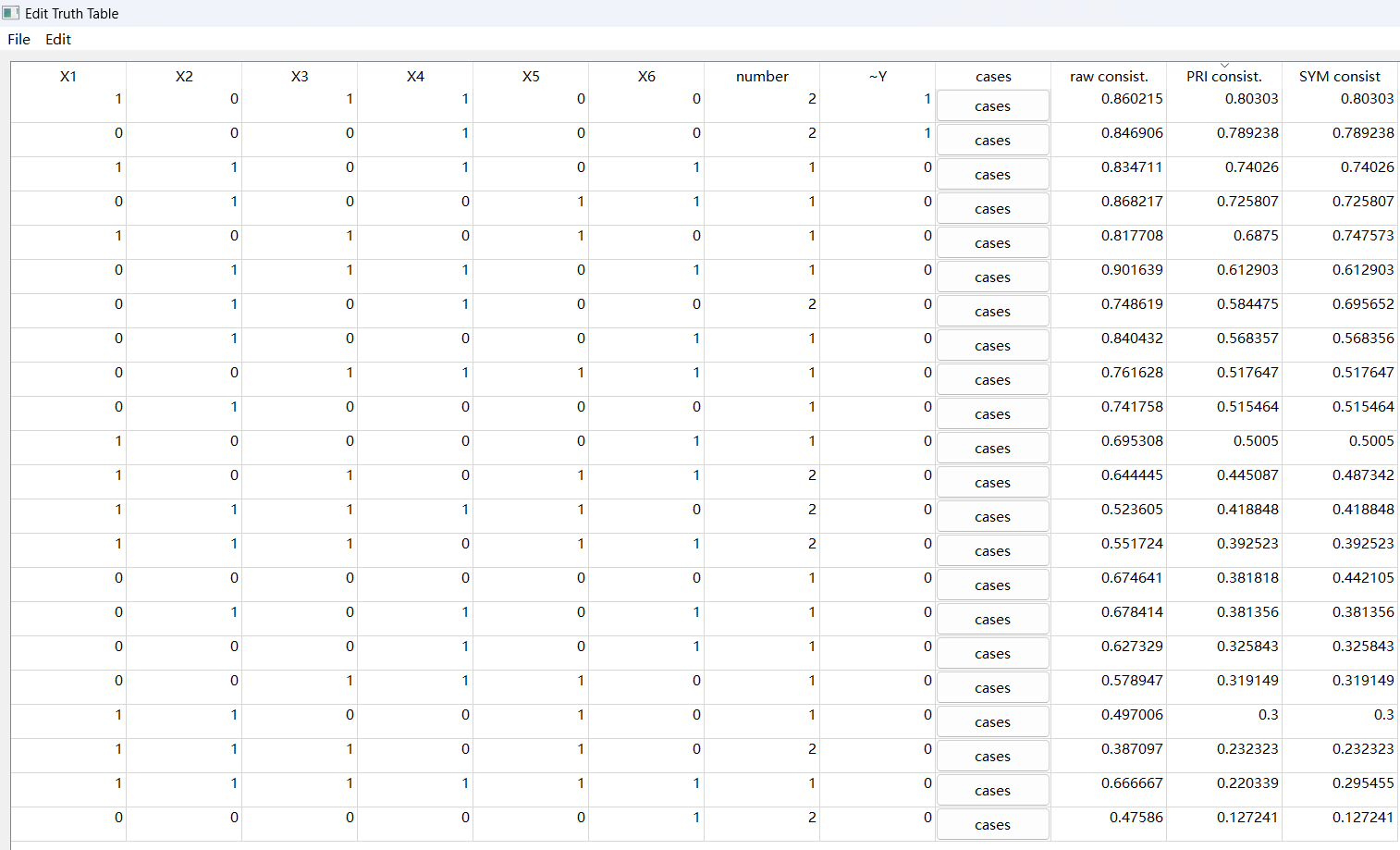


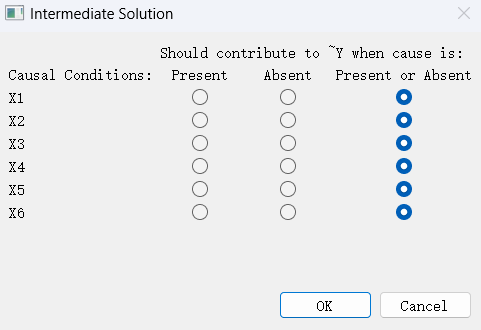


**********************

*TRUTH TABLE ANALYSIS*

**********************

File: D:/桌面/卵巢癌-校准数据-75.csv

Model: ~Y = f(X1, X2, X3, X4, X5, X6)

Algorithm: Quine-McCluskey

### --- COMPLEX SOLUTION ---

frequency cutoff: 1

consistency cutoff: 0.846906

raw unique

coverage coverage consistency

---------- ---------- ----------

~X1*~X2*~X3*X4*~X5*~X6 0.168503 0.127673 0.846906

X1*~X2*X3*X4*~X5*~X6 0.103694 0.0628645 0.860215

solution coverage: 0.231367

solution consistency: 0.841981

Cases with greater than 0.5 membership in term ~X1*~X2*~X3*X4*~X5*~X6: Guangxi (0.92,0.98), Gansu (0.72,0.98)

Cases with greater than 0.5 membership in term X1*~X2*X3*X4*~X5*~X6: Hunan (0.65,0.93), Hainan (0.54,0.93)

**********************

*TRUTH TABLE ANALYSIS*

**********************

File: D:/桌面/卵巢癌-校准数据-75.csv

Model: ~Y = f(X1, X2, X3, X4, X5, X6)

Algorithm: Quine-McCluskey

### --- PARSIMONIOUS SOLUTION ---

frequency cutoff: 1

consistency cutoff: 0.846906

raw unique

coverage coverage consistency

---------- ---------- ----------

~X2*X4*~X5*~X6 0.235904 0.235904 0.834862

solution coverage: 0.235904

solution consistency: 0.834862

Cases with greater than 0.5 membership in term ~X2*X4*~X5*~X6: Guangxi (0.92,0.98), Gansu (0.72,0.98), Hunan (0.71,0.93), Hainan (0.55,0.93)

**********************

*TRUTH TABLE ANALYSIS*

**********************

File: D:/桌面/卵巢癌-校准数据-75.csv

Model: ~Y = f(X1, X2, X3, X4, X5, X6)

Algorithm: Quine-McCluskey

### --- INTERMEDIATE SOLUTION ---

frequency cutoff: 1

consistency cutoff: 0.846906

Assumptions:

raw unique

coverage coverage consistency

---------- ---------- ----------

~X1*~X2*~X3*X4*~X5*~X6 0.168503 0.127673 0.846906

X1*~X2*X3*X4*~X5*~X6 0.103694 0.0628645 0.860215

solution coverage: 0.231367

solution consistency: 0.841981

Cases with greater than 0.5 membership in term ~X1*~X2*~X3*X4*~X5*~X6: Guangxi (0.92,0.98), Gansu (0.72,0.98)

Cases with greater than 0.5 membership in term X1*~X2*X3*X4*~X5*~X6: Hunan (0.65,0.93), Hainan (0.54,0.93)
